# Prognostic Value of Trial Round Window Stimulation for Selection of Candidates for Cochlear Implantation as Treatment for Tinnitus

**DOI:** 10.3390/jcm10173793

**Published:** 2021-08-25

**Authors:** Laure Poels, Andrzej Zarowski, Marc Leblans, Robby Vanspauwen, Joost van Dinther, Erwin Offeciers

**Affiliations:** European Institute for ORL-HNS, Sint-Augustinus Hospital, 2610 Antwerp, Belgium; laurepoels@gmail.com (L.P.); marc.leblans@gza.be (M.L.); robby.vanspauwen@gza.be (R.V.); Joost.vanDinther@gza.be (J.v.D.); erwin.offeciers@gza.be (E.O.)

**Keywords:** tinnitus, electrical stimulation, round window

## Abstract

Electrical stimulation with cochlear implants is able to significantly suppress the tinnitus sensations in 25–72% of implanted patients. Up to this point, no clear predictors for the effectiveness of tinnitus suppression with cochlear implants have been found and this substantially limits the possibility of the application of cochlear implants for this purpose. The objective of the study was to investigate if a trial electrical round window stimulation (RWS) could be used as a diagnostic tool for identifying candidates in whom electrical stimulation would be successful as treatment for tinnitus. Thirty-four patients with unilateral severe tinnitus and ipsilateral moderate to severe sensorineural hearing loss underwent a trial RWS under local anesthesia. Thirteen patients received a cochlear implant. All patients qualified for cochlear implantation on the basis of the trial RWS showed tinnitus suppression with the implant switched on. Complete or almost complete tinnitus suppression was obtained in 77% and partial in 23%. The mean tinnitus loudness reduction was 68% (VAS score reduction from 7.7 to 2.5). False negative results are estimated not to exceed 10–15%. We conclude that significant tinnitus suppression achieved during trial RWS under local anesthesia is a simple procedure allowing the efficient identification of candidates in whom electrical stimulation with a cochlear implant would be successful as treatment for intractable tinnitus.

## 1. Introduction

Tinnitus is the perception of sound that does not arise from an external source. It is described by patients as a “whistle” or “beep” sound or sounds such as “crickets” and “noise” and many others. The global prevalence of tinnitus ranges from 10 to 30%, most studies report rates between 10% and 15%. More than 40 million Europeans experience some form of tinnitus. In 0.5% of the cases, tinnitus is unbearable and associated with social, emotional and psychological sequels resulting in invalidity [1,2,3]. The exact pathophysiology of tinnitus is unknown, but in the case of associated sensorineural hearing loss, it is assumed that auditory deprivation results in pathologic reorganization and altered activity of the neural elements of the auditory pathway. There is a wide range of treatment modalities for tinnitus, but no single therapy is effective in all patients. Treatment options range from different medications and surgical approaches, external noise generators or hearing aids to psychotherapy and alternative therapies. Since the most probable cause of tinnitus is auditory deprivation, most therapies involve enrichment of the auditory environment of the patient [4]. In patients with mild to moderate hearing loss, different types of hearing aids and noise generators can be used. However, for patients with severe and profound hearing loss, the only potentially effective treatment modality is electrical stimulation, which has gained interest over the past few decades.

Electrical stimulation as a treatment for tinnitus is not new. Electrical stimulation of the hearing system in humans was first been reported by Volta in 1790. Grappengeiser discovered in 1802 that positive DC voltage applied to the external ear canal can effectively suppress tinnitus. House reported tinnitus suppression in one of the first cochlear implant (CI) patients in 1976, and Van de Heyning reported the results of cochlear implantation to treat tinnitus in patients with single-sided deafness in 2008 [5,6]. There have also been studies showing the effectiveness of promontorium/round window stimulation in suppressing tinnitus in some patients [7,8,9,10].

One main theory behind electrical stimulation as a treatment for tinnitus is that electrical stimulation of the auditory nerve can result in the recovery or limitation of the abnormal firing pattern in the affected nervous structures causing tinnitus [9]. Alleviation of tinnitus by electrical stimulation can be explained by suppression or masking. 

According to the meta-analysis of Quaranta et al., 66–86% of patients with severe to profound hearing loss present various grades of tinnitus [11]. In our own unpublished study, 66% of CI patients experienced tinnitus before implantation. In the meta-analysis of Quaranta et al., CI was able to suppress the tinnitus 46–86% of implanted patients. A more recent meta-analysis of Ramakers et al. shows that electrical stimulation with CI is able to significantly suppress the tinnitus sensations in 25–72% of implanted patients, with complete suppression in 8–45% of the patients [12]. In our own data, tinnitus suppression during electrical stimulation was observed in 66%, with complete suppression in 30% of the patients. 

However, up to this point, no clear predictors for the effectiveness of tinnitus suppression with CI have been found, and this significantly limits the possibility of application of CI for this purpose. Our previous unpublished studies were unable to show any correlations with etiology of deafness, gender and age or the stimulation strategy. This means that there is no method available for the identification of suitable candidates amongst tinnitus sufferers for CI or, even more importantly, for the identification of the significant number of patients in whom electrical stimulation for tinnitus suppression would not work at all. An adequate selection procedure is essential for improving patient selection and counseling. 

This study investigated if a trial round window stimulation (RWS) could act as a reliable diagnostic tool for identifying candidates in whom electrical stimulation would be successful as treatment for tinnitus. The objective of this study was to find out if patients who experienced tinnitus suppression during the trial round window stimulation would also experience tinnitus suppression after cochlear implantation. 

## 2. Materials and Methods

### 2.1. Study Design

Single-institution retrospective analysis. Due to ethical constrains, it was impossible to design the study as a randomized and prospective one; therefore, an alternative approach was adopted for the definition of the control group. For this purpose, we used our own data and literature data on tinnitus suppression achieved after cochlear implantation in patients who experienced tinnitus preoperatively. By comparing the percentage of the patients with positive result of the trial RWS with the percentage of the patients with CI in whom the electrical stimulation can efficiently suppress the tinnitus, an estimate of the number of the false negative results could be made. This means that the number of false negative results of RWS should be limited if the percentage of the patients with positive result of the trial RWS is similar to the own and literature data on the percentage of implanted patients in whom CI allows for efficient tinnitus suppression.

### 2.2. Subjects

The medical records of 37 patients with unilateral severe tinnitus and ipsilateral moderate to severe sensorineural hearing loss who underwent a trial RWS to evaluate the indications for CI as treatment for intractable tinnitus were retrospectively analyzed. Medical dossiers of 3 patients referred to us from other centers were not complete and were excluded from this analysis. Data of the remaining 34 patients including 10 females and 24 males with an average age of 54 years (age range from 35 to 74 years) were included. In the analyzed group of 34 patients, 13 patients (4 females and 9 males; average age 56 years; range from 35 to 74 years) received a CI (6 patients Cochlear Nucleus, 5 patients Med-El Concerto and 2 patients Advanced Bionics Hi-Focus). All implanted patients presented with an asymmetrical moderate (40–69 dBHL) to severe (>70 dBHL) sensorineural hearing loss or single sided deafness. Ipsilateral pure tone average (PTA) ranged from 68 dB HL to 120 dBHL, and the mean ipsilateral PTA was 97 dBHL. Contralateral PTA ranged from −2 dBHL to 65 dBHL, and the mean contralateral PTA was 28 dBHL. All patients in the study tried multiple therapies for alleviation of their tinnitus before the RWS. Patients with residual hearing tried hearing aids and/or the noise generators. All patients went through the Tinnitus Retraining Therapy and the Cognitive Behavioral Therapy, and some tried medication or transcranial magnetic or electrical stimulation.

### 2.3. Audiological Work-Up

After taking the medical history and clinical otologic examination, each patient was subjected to a standard audiological test battery. It included pure tone audiometry, tympanometry and a psycho-acoustic measurement of tinnitus (tinnitus characteristics, frequency and loudness, masking level and the presence of residual inhibition). CI candidates underwent speech audiometry (maximal speech discrimination scores and speech reception at 70 dBSPL were recorded) and auditory phoneme evaluation (APE) prior to implantation. Tinnitus loudness was assessed and compared preoperatively and postoperatively by using the Visual Analogue Scale (VAS). A score of 0 means that the tinnitus was inaudible or absent, and a score of 10 means that the tinnitus is extremely loud. Tinnitus loudness reduction was calculated by using the following equation: Tinnitus loudness reduction (%) = ((L0 − L1)/L0) × 100% where L0 represents tinnitus loudness preoperatively, and L1 represents tinnitus loudness postoperatively. Tinnitus loudness reduction of >30% is considered clinically relevant and successful [13]. During the follow-up, we used the following cut-off values for complete and almost complete tinnitus suppression: VAS ≤ 1 for complete suppression and VAS ≤ 2 for almost complete suppression. No tinnitus suppression was noted when the VAS score did not change by more than 1 point, and all intermediate results were defined as partial suppression. Speech comprehension after implantation was reported by using the median of the 3 best ipsilateral maximum speech reception scores and the median of the 3 best speech reception scores at 70 dB SPL during a follow-up period of 6 to 24 months. 

### 2.4. Round Window Stimulation 

Creating surgical access to the round window under local anesthesia is an easy otological procedure that can be performed by most older year ENT residents. During this procedure, the tympano-meatal flap is lifted, and a custom-made ball electrode is placed in direct galvanic contact with the round window membrane and connected to an external stimulator. We used the AxonScan stimulator (CE-labelled, produced by Medes Ltd., Aartselaar, Belgium). As reference electrodes, we used adhesive ECG electrodes that were attached to the ipsilateral and contralateral mastoid. Patients were stimulated with sinusoidal current stimuli (250–8000 Hz) and with biphasic current pulses (repetition rate 16–8000 Hz, 24 µs per phase for pulses with repetition rate of 1 kHz or higher and 32 µs per phase for pulses with repetition rate lower than 1 kHz), and the behavioral patient reactions were evaluated. The recorded parameters comprised the threshold of the auditory perceptions, the dynamic range, presence of neural adaptation, pitch discrimination and the presence of tinnitus suppression and residual inhibition. In order to evaluate the degree of tinnitus suppression during electrical stimulation, patients were asked whether the tinnitus was completely, almost completely, partially or not suppressed.

Positive advice after the trial RWS was given when the functional integrity of the auditory nerve was confirmed (low threshold of electrical stimulation for sound perception, good dynamic range and maximal comfort level with absent neural adaptation), and complete or almost complete subjective tinnitus suppression during stimulation was observed with at least one type of the signal patterns. Relatively favorable advice for CI was given to patients with partial tinnitus suppression, and negative advice was given for patients showing no tinnitus suppression during the RWS.

### 2.5. Statistical Analysis 

Descriptive statistics were used throughout this study.

## 3. Results

### 3.1. Tinnitus Analysis

In the analyzed group of 34 patients the average tinnitus pitch was 4600 Hz (range from 500 Hz to 14 kHz), and the loudness was 20 dBSL (range from 5 to 50 dBSL). The average VAS score was 7.9 (range 5–10).

### 3.2. Round Window Stimulation 

The average threshold of subjective auditory perceptions with 1 kHz sinusoidal stimulation was 196.4 µA (average maximum comfort level (MCL) 330.2 µA), with 1 kHz pulsatile stimulation 322.4 µA (average MCL 372.4 µA) and with 4 kHz pulsatile stimulation 470.4 µA (average MCL 815.7 µA). 

Out of 34 analyzed patients who underwent round window stimulation, 17 patients received positive advice for tinnitus suppression after CI (50%), 3 patients received relatively favorable advice (9%) and 14 received negative advice (41%).

Complete tinnitus suppression during RWS was observed in 13 patients (38%), almost complete in 4 patients (12%), partial suppression in 3 patients (9%) and no suppression in 14 patients (41%). Residual inhibition was present in 12 patients (35%) and absent in 22 patients (65%). Residual inhibition was generally short (duration of only a few seconds).

### 3.3. Effects of CI on Tinnitus Suppression 

A total of 13 patients received a CI. Implantation was performed in 10 out of 17 patients who received positive RWS advice, in 2 out of 3 with relatively favorable advice and in 1 patient with negative advice. The reasons for not implanting patients with positive advice were problems with reimbursement or fear for surgery. On the other hand, patients with relatively favorable and negative advices received an implant because of their wish for hearing improvement.

Complete tinnitus suppression with the speech processor switched on was reported during anamnesis by seven patients (54%), almost complete by three patients (23%) and partial suppression by three patients (23%). 

**Figure 1:**
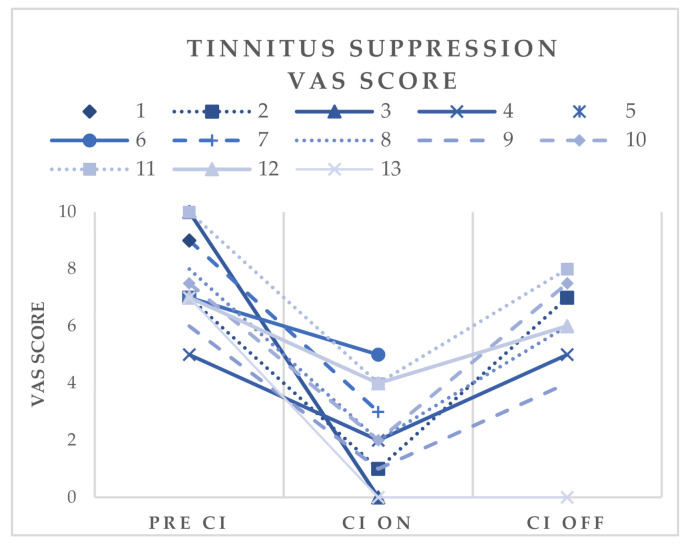
Tinnitus suppression. Evolution of VAS scores in 13 implanted patients. Pre CI = before cochlear implantation; CI on = speech processor of cochlear implant switched on; CI off = speech processor of cochlear implant switched off.

The VAS score was used to evaluate the effect of electrical stimulation on tinnitus loudness. Complete suppression (VAS ≤ 1) was observed in four patients (31%), and (almost) complete suppression (VAS ≤ 2) was observed in seven patients (54%). The preoperative VAS scores were compared with the postoperative VAS scores in the ON or OFF condition of the CI. The average preoperative VAS score in 13 patients who received a CI was 7.7 (range 5 to 10), the postoperative score with the speech processor switched ON was 2.5 (range 0–6) and the postoperative score with the speech processor switched OFF was 5.1 (range 0–8). Figure 1 shows the evolution of the VAS scores in each of 13 implanted patients. Tinnitus loudness reduction while using the speech processor ranged from 29% to 100% with a mean tinnitus loudness reduction of 68%. All but one patient with positive or relatively favorable advice had significant and clinically relevant tinnitus loudness reduction while using the speech processor.

Out of twelve patients with positive or relatively favorable advice of the RWS, eleven are permanent users of their implants, and one patient is only an occasional user (this patient shows only partial tinnitus suppression with CI and his preoperative VAS score was five). One patient with the negative advice of the RWS became a non-user. This patient shows only partial tinnitus suppression and additionally suffers from hyperacusis and pain sensations with the implant switched on. 

In 11 of 13 implanted patients (85%), the degree of tinnitus suppression during anamnesis was exactly as predicted by round window stimulation or better. In two patients who showed complete tinnitus suppression during the RWS, one had almost complete suppression, and the other only had partial tinnitus suppression with CI. 

No complications after round window stimulation were encountered.

### 3.4. Results of CI on Speech Comprehension

Amongst the 13 implanted patients, preoperative speech comprehension was as follows: mean maximum ipsilateral speech discrimination scores and mean ipsilateral speech discrimination scores at 70 dBSPL without hearing aid were 13% and 0%, respectively. 

Postoperatively, the mean maximum ipsilateral speech discrimination scores and mean ipsilateral speech discrimination scores at 70 dBSPL with the CI switched on were 78% and 75%, respectively. Auditory phoneme evaluation showed excellent average detection and discrimination scores (detection 15/15; discrimination 20/22). 

## 4. Discussion

Our results show that electrical stimulation by CI was able to suppress the tinnitus sensations in all patients selected on the basis of the positive or relatively favorable result of the RWS test. Complete or almost complete tinnitus suppression was obtained in 77% and partial suppression in 23%. The mean tinnitus loudness reduction was 68% (VAS score reduction from 7.7 to 2.5). All but one patient had significant and clinically relevant tinnitus reduction after CI using the speech processor (tinnitus reduction >30%). One patient with tinnitus loudness reduction of less than 30% was one of two patients who showed only partial tinnitus suppression during the RWS; thus, this result was as expected. Complete tinnitus suppression after CI was reported by seven (54%) of the patients during anamnesis, while based on VAS scores (VAS ≤ 1), complete suppression was observed in four (31%). Almost complete or complete tinnitus suppression was reported by 10 (77%) of the patients on anamnesis, while based on VAS-scores (VAS ≤ 2), (almost) complete suppression was observed in seven patients (54%). We noticed that the VAS scores relating to tinnitus loudness often do not match the degree of tinnitus suppression reported by patients during anamnesis. One patient reported persistent significant tinnitus relief (tinnitus reduction >30%) even when the CI was switched off. Except for this one patient, the alleviation of tinnitus by electrical stimulation through a CI seems to work mainly by delivering an audible stimulus and changing the activity pattern of the ipsilateral acoustic pathways. 

In the current study, 20/34 patients (59%) received positive or relatively favorable advice for CI on basis of the results of RWS. According to the recently published meta-analysis of Ramakers et al., since electrical stimulation is able to efficiently suppress the tinnitus in 25–72% (66% in our own data) of the patients with CI, we can assume that the number of false negative results should not exceed 10–15% [12]. This means that, in the worst case, using the RWS as the diagnostic test would exclude 10–15% of the patients who could still benefit from CI for tinnitus suppression. We consider this as a reasonable tradeoff for ensuring good results in patients who were qualified for CI on basis of the RWS.

To date, there are no studies reporting on round window stimulation as a diagnostic test prior to cochlear implantation, but there are some studies reporting on round window stimulation for tinnitus suppression ([9,10]. In the study of Wenzel et al., three patients with severe to profound hearing loss/deafness and ipsilateral tinnitus were implanted with a non-penetrating round window electrode. All patients had a benefit (THI score improvement) from the electrical stimulation for their tinnitus.

The objective of this study was to investigate if a trial round window stimulation could serve as a reliable diagnostic tool for identifying candidates in whom electrical stimulation would be successful as treatment for tinnitus. In 11 of 13 implanted patients (85%), the degree of tinnitus suppression was exactly as predicted by round window stimulation or better. Only two patients with complete tinnitus suppression during round window stimulation had either only partial or almost complete suppression postoperatively. The non-user was implanted in spite of the negative advice related to the possibility of tinnitus suppression and received the implant in order to improve his hearing and speech discrimination.

We recognize the limitations of this retrospective study because of the small study population and the absence of a control group. Due to ethical constrains, it was impossible to design the study as a randomized and prospective one. CI for tinnitus suppression is not reimbursed in Belgium, and patients have to pay for the implants themselves. Since electrical stimulation is unable to suppress tinnitus in up to 50% of the patients, it was considered unethical to allow all patients undergo CI and to pay for the implants when a significant percentage of patients would not benefit from it. However, exclusion of the patients with negative results of the RWS from CI could introduce a number of false negative results, i.e., patients disqualified for CI who could still obtain efficient tinnitus suppression with an implant. As shown above, the number of false negative results is estimated not to exceed 10–15%. On the other hand, false positive results (placebo effect) cannot be excluded in this relatively small study population and require confirmation in larger cohorts.

Despite this, the results in this study show that round window stimulation can act as an efficient diagnostic tool for selecting candidates with sensorineural hearing loss and tinnitus who would benefit from CI for both speech comprehension and tinnitus reduction.

## 5. Conclusions

Round window stimulation is a reliable and safe diagnostic tool allowing for the identification of candidates in whom electrical stimulation would be successful as treatment of tinnitus. Development of a diagnostic tool with reliable predictive value could not only improve the results of CI in patients with tinnitus in the deaf ear but could also result in development of an implantable electrical stimulator for tinnitus suppression in patients with usable hearing. 

## Data Availability

The data presented in this study are available upon request from the corresponding author. The data are not publicly available due to privacy and ethical restrictions.
